# Effects of voluntary removal of ractopamine hydrochloride (Optaflexx) on live performance and carcass characteristics of beef steers

**DOI:** 10.1093/tas/txab047

**Published:** 2021-03-09

**Authors:** Phillip J Rincker, Janet B Allen, Matt Edmonds, Michael S Brown, John C Kube

**Affiliations:** 1 Elanco, Greenfield, IN 46140, USA; 2 Johnson Research, LLC, Parma, ID 83660, USA

**Keywords:** β-adrenergic agonist, extra-label use, growth performance, voluntary removal

## Abstract

There is a lack of consistency across the globe in how countries establish tissue ractopamine residue limits and which residue limits are applied to various tissues, particularly for edible noncarcass tissues. Therefore, some U.S. beef slaughter organizations have recommended a 48-h voluntary removal of ractopamine before slaughter to meet residue requirements of specific export countries and maintain international trade. Our objective was to assess the impact of voluntary removal of ractopamine hydrochloride (Optaflexx; Elanco, Greenfield, IN) up to 8 d before slaughter on growth performance and carcass characteristics. Crossbred beef steers (60 pens of 10 animals/pen) with an initial shrunk body weight (**BW**) of 611.8 ± 10 kg SEM were fed one of six treatments over 42 d. Treatments included a control that did not receive ractopamine, on-label use of ractopamine (0-d withdrawal), and 2, 4, 6, or 8 d of voluntary removal of ractopamine from feed before slaughter. The start of ractopamine feeding (30.1 mg/kg of diet dry matter for 32 d) was staggered, so that blocks could be slaughtered on the same day. Dry matter intake was decreased by 0.5 kg/d when ractopamine was fed with a 0-d withdrawal (*P* = 0.04) compared with the control, but was not altered (*P* = 0.56) as the duration of ractopamine removal increased from 0 to 8 d. Final BW, total BW gain, and average daily BW gain were increased by feeding ractopamine with a 0-d withdrawal (*P* = 0.09) compared with the control, but these variables decreased in a linear manner (*P* = 0.10) as the duration of removal increased from 0 to 8 d. Gain efficiency was improved by 15% (*P* < 0.01) by feeding ractopamine with a 0-d withdrawal compared with the control, and gain efficiency decreased linearly (*P* = 0.06) as the duration of ractopamine removal increased. Approximately 2/3 of the increase in gain efficiency remained after 8 d of removal. Hot carcass weight was increased by 6 kg (*P* = 0.02) by feeding ractopamine with a 0-d withdrawal compared to the control. Measured carcass characteristics were not altered by ractopamine feeding or by removal before slaughter (*P* ≥ 0.24). The consequences of voluntary removal of ractopamine up to 8 d before slaughter were a linear decrease in live BW gain (0.64 kg/d), poorer gain efficiency, and numerically lighter carcass weight.

## INTRODUCTION

Ractopamine hydrochloride is classified as a β-adrenergic agonist and was first approved for use in beef cattle as a feed additive in the United States (Optaflexx; Elanco) by the Food and Drug Administration in 2003 (FDA, 2003). Ractopamine has been widely fed to feedlot steers and heifers since that time to increase the rate and efficiency of weight gain. A key part of feed additive approval in the United States is determining the need to establish residue tolerances and any necessary withdrawal period before slaughter to ensure human safety. Optaflexx was approved with a 0-d withdrawal; the 0-d withdrawal is formally defined as 3 to 12 h of ractopamine (and feed) withdrawal before slaughter because this duration of time encompasses expected transport and lairage time of cattle at the slaughter plant.

The Center for Veterinary Medicine of the U.S. Food and Drug Administration establishes residue tolerances in the United States for the appropriate target tissues (muscle, fat, liver, or kidney) from food animal species for which a feed additive is approved and may establish tolerances for all four of these primary tissues. Many export countries establish their own maximum residue limits or follow the Codex recommendations for edible animal tissues imported from the United States (Codex Alimentarius, 2018). Globally, there is inconsistency around how residue limits are established and which residue limits are applied to various tissues. For example, Japan currently follows the Codex residue limits for ractopamine of 40, 90, 10, and 10 μg/kg for liver, kidney, muscle, and fat, respectively, and applies the liver residue limit (40 μg/kg) to all other tissues (e.g., edible noncarcass tissues; FSIS, 2021). South Korea also currently applies the Codex residue limits, but applies the muscle residue limit (10 μg/kg) to all other tissues (FSIS, 2021). Thus, some U.S. beef slaughter organizations have recommended a 48-h voluntary removal of ractopamine before slaughter to meet residue requirements of specific export countries.

The FDA enforces adherence to the approved label for feed additives in the United States. However, some cattle feeders may be required to remove ractopamine for several days before slaughter to maintain cattle marketing competition. These cattle feeders would benefit from knowledge of any growth performance consequences that may accompany removal. The data of Bryant et al. (2020) suggest that the added weight gain from feeding ractopamine was maintained through 7 d after removal. These data seem in contrast with pharmacokinetic data that have established that the half-life of orally administered ractopamine under steady-state conditions is approximately 24 h in beef steers and heifers (Elanco, unpublished observations) and to results indicating that ractopamine fed to beef steers and heifers at 45 mg/kg of dry matter is no longer detectable in skeletal muscle or subcutaneous fat by 48 h after ractopamine removal (FDA, 2003). However, the rate at which metabolic processes in muscle and adipose return to baseline after ractopamine removal is unknown. Data from swine further indicate that the entire mass of added weight gain from feeding ractopamine for 25 d is completely lost after as little as 10 d of ractopamine removal (Johnston et al., 2007). Additional data are needed to characterize the growth performance response by beef cattle during removal of ractopamine. Our objective was to assess the impact of voluntary removal of ractopamine hydrochloride up to 8 d before slaughter on growth performance and carcass characteristics.

## MATERIALS AND METHODS

### Animal Selection, Randomization, and Acclimation

The study was conducted by a research facility in Idaho in January and February 2019. Animal care procedures followed FASS (2010), and the study was conducted under an approved animal care protocol (EIAC-0988). The entry of beef into the food chain from cattle fed ractopamine outside of label clearances was possible by a food use authorization (I-004736-D-0052-OT) by the FDA. Conservative replication needs were determined based on previous experiments to detect a difference of 5.4 kg of carcass weight (i.e., control vs. 0-d withdrawal) with power = 0.80 and α = 0.05.

English and English × Continental beef steers (*n* = 600; 60 pens of 10 animals/pen) were used in a randomized complete block design. Steers were selected from the available population previously being fed a finishing diet at a commercial feedlot. Selection was based on similarity in live body weight (**BW**), days on feed, and phenotype, and steers were then transported to the study site prior to day −70 (day 0 = slaughter). On approximately day −68, all steers were individually weighed using a single-animal chute scale (Model IQ 335, Rice Lake Weighing Systems, Rice Lake, WI), individually identified, existing implants were removed, and steers were implanted with Component TE-S (24 mg of estradiol and 120 mg of trenbolone acetate; Elanco). This individual BW was used to stratify eligible cattle by increasing BW and establish weight blocks from lightest to heaviest. Random sequences of the six treatments were then applied successively to individuals within block to the stratified BW. Cattle were then sorted according to the block and treatment assignment into study pens on approximately day −62 as they exited the chute. Adjacent pens were designated as blocks and pens within block were randomized to treatments. Pens (22.9 m × 30.5 m) were outdoor, soil-surfaced without shelter, and contained 7.6 m of concrete fenceline bunk and 0.9 m of water trough space. Logistical limitations of cattle handling and shipping capacity required that study cattle be divided into two harvest groups; harvest groups 1 and 2 (five blocks each) were determined based on block location so that contiguous pens and pens in adjoining alleys formed a group. Subsequent study events occurred on consecutive days for these two harvest groups. Cattle were allowed the subsequent 20 d to acclimate to the facility and the basal diet.

### Treatments

On day −42, a pen live BW was collected before feeding using a 45,000-kg capacity pen scale (Model WI-130, Avery Weigh-Tronix LLC, Fairmont, MN; 3.66 m × 21.34 m), and this served as initial BW for the study. Treatments began with feed delivered after the weighing event that morning. Treatments included a control diet that did not contain ractopamine (Table 1), and a ractopamine-containing diet (30.1 mg of ractopamine/kg of dry matter) that was fed for 32 d and then removed from the diet for 0, 2, 4, 6, or 8 d before slaughter. The term withdrawal will be used throughout to refer to an FDA-approved use according to the label (i.e., 0-d withdrawal), whereas voluntary ractopamine removal will be used to denote extra-label use.

**Table 1.** Ingredient and chemical composition of the basal diet dry matter^1^

Ingredient composition	% of dry matter
Dry-rolled corn	31
High-moisture corn	17
Dry-rolled wheat	10
Earlage, corn	18
Dry distiller’s grains	3
Supplement^2^	5
Condensed distiller’s solubles	5
Tallow	3
Wheat straw	3
Alfalfa hay	5
Assayed components	
Dry matter	65.2
Crude protein	14.4
Calcium	0.8
Phosphorus	0.4
Calculated components	
Neutral detergent fiber	17.6
Acid detergent fiber	8.9
Ether extract	6.6
NE_m_, Mcal/kg^3^	2.15
NE_g_, Mcal/kg^3^	1.50

^1^Assayed composition from six weekly composite samples that were derived from three subsamples of a randomly chosen batch each week.

^2^Liquid supplement contained (dry basis): 74% crude protein (67% of crude protein as nonprotein nitrogen), 0.9% ether extract, 13.2% calcium, 0.1% phosphorus, 0.6% magnesium, 2.8% potassium, 0.4% sulfur, 447 ppm manganese, 1,318 ppm zinc, 259 ppm copper, 4.7 ppm cobalt, 11.7 ppm iodine, 4.7 ppm selenium, 147,073 IU/kg of vitamin A, 14,773 IU/kg of vitamin D, 163 IU/kg of vitamin E, 977 mg/kg of monensin, and 197 mg/kg of tylosin.

^3^Calculated from tabular values for individual ingredients based on NRC (2000). NE_m_ = net energy for maintenance; NE_g_ = net energy for gain.

Each treatment other than the control received the control diet for the appropriate length of time before ractopamine was fed (Table 2). The beginning of ractopamine feeding was staggered as per treatment to allow a common slaughter day for all treatments within a block and harvest group, and the study comprised 42 d for all treatments. Both diets contained 48.5 mg of monensin/kg of dry matter (Rumensin 90; Elanco) and 9.8 mg of tylosin/kg of dry matter (Tylan 100; Elanco).

**Table 2.** Duration of time that different diets were fed to steers fed the control diet and steers receiving 0, 2, 4, 6, or 8 d of voluntary ractopamine removal before a common day of slaughter across treatments^1^

Duration of ractopamine removal, d	Initial control diet duration, d	Ractopamine diet duration^2^, d	Control diet duration during removal, d	Total study duration, d
Control	42	0	0	42
0	10	32	0	42
2	8	32	2	42
4	6	32	4	42
6	4	32	6	42
8	2	32	8	42

^1^Control cattle received the control diet without ractopamine for the entire 42-d study.

^2^The ractopamine diet contained 30.1 g of ractopamine/kg of dry matter.

Final BW was determined on a pen basis before feeding on the day cattle were shipped to slaughter. The actual duration from the last feeding until slaughter (i.e., withdrawal) was 17.5 to 21 h for harvest group 1 (five blocks) and 22.5 to 26 h for harvest group 2 (five blocks). All animal scales were certified before the study and validated before each use. The single-animal chute scale was set to a resolution of ±0.45 kg, and the platform pen scale was set to a resolution of ±4.5 kg. Acceptable accuracy for all scales before recalibration was required was ±5% of the theoretical weight.

### Feeding and Slaughter Procedures

Bunks were assessed daily at approximately 0630 h. Cattle were fed once daily to appetite, beginning at 0700 h and ending by 0900 h, during acclimation and throughout the study by adjusting feed deliveries to maintain traces of refused feed before feeding. Refused feed was weighed on weigh days and as needed throughout the study and dry matter determined (>12 h at 100°C). Mixing procedures to ensure homogeneity of mixed feed were confirmed before the start of the experiment, and mixer scales were validated before use each day. The tolerance for acceptable batching accuracy for ingredients other than ractopamine in a given batch was ±13.6 kg. A stationary mixer (Kirby model 705; 20.0 m^3^ capacity) was used to prepare the control diet, and this feed was transferred into two trailer-mounted mixers (Kirby 475; 13.5 m^3^ capacity) for final preparation and delivery. One trailer mixer was dedicated to the control diet, and the second trailer mixer was dedicated to the ractopamine diet. Product containing ractopamine was weighed to the nearest 0.45 g (scale validated before and after daily use) and added to the microingredient machine, and machine contents were added to the appropriate mixer via a water slurry; the water slurry only was added from the empty microingredient machine to the appropriate mixer dedicated to the control diet to maintain equal diet dry matter. The stationary mixer scale was set to a resolution of ±4.5 kg, and the trailer mixer scales were set to a resolution of ±2.3 kg. Before and after feeding each day, the trailer-mounted mixers were cleaned of medicated feed residues with a flush batch of non-medicated feed and the flush feed was discarded.

Cattle were transported to a commercial slaughter facility in the northwest United States by harvest group on consecutive days; harvest group 1 was loaded and shipped between 1139 and 1330 h and harvest group 2 was loaded and shipped between 1056 and 1230 h. Experienced staff collected ear tag number, plant ID, and carcass sequence and applied a unique carcass ID after head removal to track carcasses throughout the process. Data collected by research staff on the kill floor included hot carcass weight, instances of excessive trim (> approximately 9 kg), and railed carcasses. Ribfat thickness was measured by research staff on chilled carcasses at three-fourth of the length of the longissimus dorsi muscle from the chime bone end, whereas remaining carcass cooler measures of marbling score and longissimus dorsi muscle area were obtained from plant data (measured by instrument grading) matched to the unique carcass ID applied.

### Laboratory Analyses

Dry matter intake (**DMI**) was the difference between dry feed delivered and dry refused feed. Diet dry matter was determined weekly by oven drying (>12 h at 100°C) for both diets. Diet samples were collected once per week from a randomly chosen batch of each diet (control and ractopamine) for both nutrient and ractopamine analysis. A composite sample (approximately 2 kg) for each analysis × diet was generated by combining the three subsamples collected from each 1/3 of the chosen batch. Composite samples were stored at −20 °C until submission. This process was repeated for the six consecutive weeks of the study such that two composite samples per week (one per treatment) were submitted on dry ice for ractopamine analysis (Eurofins; Greenfield, IN) by HPLC techniques (CVM, 1999) and another two composite samples per week were submitted on dry ice for crude protein (AOAC, 2019; method 990.03), calcium, and phosphorus (AOAC, 2019; methods 968.08 and 985.01) analysis by MVTL Laboratories (New Ulm, MN).

### Statistical Analysis

All data were analyzed as a randomized complete block design using the Mixed procedures of SAS (version 9.3; SAS Institute, Cary, NC) and pen served as the experimental unit. Performance data are presented with dead animals excluded. One animal receiving the 4-d removal and one animal receiving the 8-d removal died during the study; no animals met the removal criteria. Initial BW was used as a covariate for final BW and hot carcass weight, but did not remain in the model for other variables (*P* > 0.10). The model included the random effect of block, harvest group, and block within harvest group, and the fixed effect of treatment. Means were only separated following a protected *F*-test (*P* = 0.20, two tailed) into the contrast of the control versus on-label ractopamine feeding (0-d withdrawal), and the linear and quadratic effects of duration of removal (i.e., 0 to 8 d). Statistical significance was declared at α ≤ 0.10 and tendencies at α ≤ 0.15. The Reg procedures of SAS were used to regress response variables against days of ractopamine removal.

## RESULTS

### Live Performance

Diet samples confirmed that ractopamine was fed at 27.1 mg/kg of dry matter on average (from six weekly samples) and that ractopamine was not detectable in the control ration (<2.5 mg/kg as-fed limit of measurement; from six weekly samples). Actual ractopamine intake averaged 271 mg/d. Final BW (Table 3) was increased 5.8 kg by feeding ractopamine with a 0-d withdrawal (*P* = 0.09) compared with the control, and final BW decreased in a linear manner (*P* = 0.10) as the duration of removal increased from 0 to 8 d. Likewise, total BW gain and daily BW gain were increased by feeding ractopamine with a 0-d withdrawal (*P* = 0.09) and both measurements of BW gain decreased in a linear fashion (*P* = 0.10) as the duration of ractopamine removal increased from 0 to 8 d. The change in predicted total BW gain during ractopamine removal was determined by regressing BW gain and days of removal (0, 2, 4, 6, and 8 d; i.e., excluding the control) and was described by the equation, *y* (total BW gain during removal, kg) = 66.80 – 0.637 × (days of removal; *R*^2^ = 0.55). After 8 d, approximately 68% of the predicted BW gain advantage from feeding ractopamine was lost.

**Table 3.** Effects of ractopamine removal on growth performance and carcass characteristics of feedlot steers

	Duration of ractopamine removal^1^								
Item	Control	0	2	4	6	8	SEM^2^	*P* > *F*^3^	Polynomial and OSL^4^
Pens	10	10	10	10	10	10	—	—	—
Days on study	42	42	42	42	42	42	—	—	—
Initial BW, kg	611.8	609.8	613.7	610.8	611.4	613.0	10.0	0.45	—
Final BW^5^, kg	671.1^a^	676.9^b^	680.3	675.2	674.0	673.7	2.4	0.16	L, 0.10
BW gain, kg	59.3^a^	65.2^b^	68.5	63.4	62.2	62.0	2.4	0.15	L, 0.10
DMI^6^, kg/d	10.38^a^	9.88^b^	10.09	9.88	9.96	9.85	0.2	0.19	Q, 0.56
Daily BW gain, kg/d	1.41^a^	1.55^b^	1.63	1.51	1.48	1.48	0.06	0.15	L, 0.10
Gain efficiency, g/kg	136^a^	157^b^	162	152	149	150	5	0.01	L, 0.06
Hot carcass weight^5^, kg	404.7^a^	410.7^b^	411.7	409.0	407.9	408.9	1.7	0.09	L, 0.18
Dressed yield, %	62.8	63.2	63.0	63.1	63.0	63.2	0.2	0.58	—
Marbling score^7^	538	518	526	523	523	517	9	0.51	—
LMA^8^, cm^2^	89.2	92.4	92.6	92.4	93.0	93.7	1.5	0.24	—
Fat depth, cm	1.43	1.38	1.47	1.41	1.42	1.42	0.05	0.73	—

^1^Control cattle did not receive ractopamine. Ractopamine was removed for 0 (on-label use), 2, 4, 6, or 8 d before slaughter.

^2^Standard error of the least square means.

^3^Significance level of the analysis of variance *F*-test. Means were not separated unless *P* > *F* was ≤0.20 (two tailed).

^4^OSL = observed significance level for linear (L) and quadratic (Q) polynomials for the duration of removal (0 to 8 d). A “—” is used to indicate that means were not separated due to *P* > *F* greater than 0.20.

^5^Initial BW used as a covariate.

^6^DMI = dry matter intake.

^7^Small = 400 to 499, modest = 500 to 599, etc.

^8^LMA = longissimus dorsi muscle area.

^a,b^Means differ for the contrast of the control vs. 0-d withdrawal (*P* < 0.10).

Steer DMI was decreased by 0.5 kg/d when ractopamine was fed with a 0-d withdrawal compared to the control (*P* = 0.04), but DMI was not altered (*P* = 0.56) as the duration of ractopamine removal increased (Table 3). Thus, gain efficiency was improved by 15% (*P* < 0.01) by feeding ractopamine with a 0-d withdrawal compared to the control, and gain efficiency linearly decreased (P = 0.06) as the duration of ractopamine removal increased from 0 to 8 d. This response was driven by the linear decrease in BW gain as the duration of removal increased.

### Carcass Characteristics

Steer dressing percentage (Table 3) was not altered by feeding ractopamine with a 0-d withdrawal compared with the control, nor did increasing the duration of ractopamine removal alter dressing percentage (*P* = 0.58). However, steer hot carcass weight was increased by 6 kg (*P* = 0.02) by feeding ractopamine with a 0-d withdrawal compared with the control. Though not different (*P* = 0.18, linear), hot carcass weight numerically decreased as the duration of ractopamine removal increased and followed a pattern similar to that of live BW and BW gain. The numeric change in predicted hot carcass weight during ractopamine removal was determined by regressing hot carcass weight and days of removal (0, 2, 4, 6, and 8 d; i.e., excluding the control) and was described by the relationship, *y* (hot carcass weight, kg) = 411.13 − 0.370 × (days of removal; *R*^2^ = 0.59). Carcass marbling score, longissimus muscle area, and ribfat thickness were not altered by ractopamine feeding (*P* ≥ 0.24).

## DISCUSSION

Ractopamine, a β _1_-adrenergic agonist, increases protein synthesis, but has little effect on protein degradation and only small effects on adipose accretion (Johnson et al., 2014). In addition, a decrease in energy demand by splanchnic tissues when feeding ractopamine (Koontz et al., 2010) probably provides key energy to power anabolism by skeletal muscle.

The direction and magnitude of the performance response by steers receiving ractopamine with a 0-d withdrawal in the present study are generally consistent with the literature. We deem the of results of meta-analyses of ractopamine efficacy far more useful for robust discussion than choosing a subset of individual ractopamine studies conducted over the past 17 years. The meta-analyses confer the advantage of reflecting point estimates and confidence intervals that are a product of experiments conducted under a variety of conditions (season, climate, cattle genetic potential, etc.) across time.

Pyatt et al. (2013) and Elanco (2018) reported an analysis of 32 steer experiments (26,000 steers) conducted across 12 states in 3 countries that were fed 0, 100, 200, or 300 mg of ractopamine/d with a 0-d withdrawal. Steers fed 300 mg of ractopamine/d had similar dry matter intake, heavier final BW (10 kg), heavier carcass weight (9 kg), and improved gain efficiency (16%) compared with control steers. Elanco (2018) noted that cattle fed ractopamine tended to have slightly leaner carcasses with greater muscling than control steers; however, that response was not observed in the present study. Furthermore, the 95% confidence interval of the effect of ractopamine on hot carcass weight for cattle was 7.7 to 10.6 kg for cattle fed 300 mg/d, 5.2 to 7.1 kg for cattle fed 200 mg/d, and 2.6 to 3.5 kg for cattle fed 100 mg/d. Thus, the carcass weight response in the present study (6 kg) was below the confidence interval for 300 mg, and well within the confidence interval for 200 mg/d. This outcome aligns with the actual ractopamine dose of 271 mg/d in the present study. The expected mean live BW gain based on the data of Elanco (2018) would be 10.2 kg at 300 mg/d, 6.8 kg at 200 mg/d, and 3.4 kg at 100 mg/d. Thus, our observed BW gain (5.9 kg) between feeding ractopamine with a 0-d withdrawal and the control was slightly lower than the expected mean for steers fed 200 mg/d in this meta-analysis.

Lean et al. (2014) evaluated 40 to 54 ractopamine studies, depending on the response variable. These authors reported that cattle fed ractopamine had a heavier live BW (7.6 kg; lower 95% confidence = 5.6 kg, upper = 9.6 kg), more rapid average daily gain (**ADG**; 0.193 kg/d), improved gain efficiency (0.018 kg/kg), heavier carcass weight (6.2 kg; lower 95% confidence = 4.6 kg, upper = 7.8 kg), and similar DMI to control cattle. Thus, these data also reasonably correspond to the outcomes evident in the current study for cattle fed ractopamine with a 0-d withdrawal compared with control cattle.

A previous report (Bryant et al., 2020) suggests that the added weight gain from feeding ractopamine with a 0-d withdrawal was maintained for at least 7 d after removal. In fact, a quadratic increase in carcass weight occurred, with peak carcass weight at 4 d of removal. A key concern with these data is the low probability based on the comprehensive analyses discussed previously of the small magnitude of improvement these authors observed from feeding ractopamine with a 0-d withdrawal. These authors reported that steers fed ractopamine with a 0-d withdrawal (314 mg for 33 d) gained 3 kg more BW than the control steers and carcasses from steers fed ractopamine according to the label were 4 kg heavier. The data summaries discussed previously suggest that the small weight gain responses observed by Bryant et al. (2020) for cattle fed ractopamine with a 0-d withdrawal would be observed less than 5% of time for the dose fed (Lean et al., 2014) or align more closely with a dose of 100 mg/d than the dose fed (Elanco, 2018). The data from the present study indicate a different conclusion about the impact of ractopamine removal on growth performance (linear decrease in BW gain and gain efficiency up to 8 d of removal) than the data of Bryant et al. (2020; no change in final BW or ADG up to 7 d of removal, quadratic increase in carcass weight). Thus, additional estimates of the impact of ractopamine removal on growth performance are warranted.

The present study demonstrates that voluntary removal of ractopamine was accompanied by a linear decrease in live BW and gain efficiency, and numerically lighter carcass weight as removal increased up to 8 d. This outcome would require cattle previously fed ractopamine to have much poorer performance following removal of ractopamine than their control contemporaries. For example, cattle for which ractopamine was removed for 8 d before slaughter in the present study had 3.2 kg less BW gain (or −0.4 kg/d of removal) than those with 0-d of withdrawal, whereas the increase in ADG when ractopamine was fed with a 0-d withdrawal (no removal) was + 0.14 kg/d. The predicted effect of a 48-h removal as has been requested by some U.S. beef slaughter organizations would be a minimum of 1.3 kg of BW/animal compared with feeding ractopamine with a 0-d withdrawal based on the present study. In practice, the actual impact of any voluntary removal is expected to be larger than the minimum estimated from the present data because diet changes are commonly made once/week in production to avoid undue complexity in feeding logistics. Thus, ractopamine removal would functionally span between approximately 2 and 7 d in production to comply with a 48-h removal. We were unable to identify in vitro or in vivo data in the literature that illuminate possible biological explanations for the observed growth response during removal. However, other lines of evidence provide support for the observations reported by the present study.

Removing dietary ractopamine from pig diets for more than a 0-d withdrawal (also an extra-label use in swine) is accompanied by a precipitous decline in growth rate. Neill et al. (2010) reported that ADG by pigs fed ractopamine for 21 d was 12% greater than control pigs, but then 8% lower than control pigs over the next 14 d when ractopamine was not fed. Over the 56-d feeding period, ADG was not altered when pigs were fed ractopamine the first 21 d compared with control pigs (0.95 vs. 0.94 kg/d, respectively). Johnston et al. (2007) observed that growth rate by pigs fed ractopamine for 25 d followed by a control diet for 10 d was not different from those fed a control diet for the entire duration (1.02 vs. 1.03 kg/d, respectively). However, feeding ractopamine the last 25 d, after feeding the control diet the first 10 d, resulted in greater ADG (mean = 1.12 kg/d). Collectively, these data demonstrate that the growth advantage from feeding ractopamine (for 21 to 25 d) to pigs is completely offset by a reduction in growth rate during 10 to 14 d of removal. Based on the live BW regression in the present study, approximately 12 d of ractopamine removal would be required for complete loss of the added BW gain from feeding ractopamine to steers.

The growth performance outcomes in the present study also seem to align with the demonstrated underlying biology of ractopamine metabolism by cattle. Elanco (unpublished observations) characterized the pharmacokinetics of ractopamine in steers and heifers (*n* = 8/sex). Cattle were orally dosed with 200 mg of ractopamine/d (0.4 mg/kg of BW, mean BW = 516 kg) for 7 d. The four isomers of ractopamine reached peak circulating concentrations (0.5 to 0.8 ng/mL) at 168 h after the initial dose. The half-life of isomers ranged from 23 to 28 h, whereas the mean retention time was 10 h. Thus, one would expect approximately 25, 6, 1.5, and 0.5% of steady-state circulating ractopamine remaining after 2, 4, 6, and 8 d of removal. Given that the minimum effective dose of ractopamine in cattle is 100 mg/d (e.g., FDA, 2003), an animal previously fed 300 mg/d should effectively have less than the minimum stimuli between 1 and 2 d of removal.

The FDA (2003) reported ractopamine residue depletion data supporting Optaflexx approval in the Freedom of Information summary. Cattle received an intraruminal dose equivalent to 45 mg of ^14^C-ractopamine HCl/kg of dry matter for 4, 7, or 10 d (1.5× of the maximum approved dose). At 12 h after the last dosing, cattle were slaughtered and muscle, fat, liver, and kidney samples were assayed for radioactivity. Thus, these residues would reflect metabolites as well as the parent molecule (i.e., total ractopamine). The residues in muscle and fat, the target tissues of the anabolic effects of ractopamine, were below the limit of detection at all durations tested. In a second study, total residues were examined after 0, 2, 4, or 7 d of removal of 45 mg of ^14^C-ractopamine HCl/kg of dry matter. Muscle and fat residues were below the limit of detection at all removal durations longer than 0 d. The residue depletion data clearly substantiate the 24-h half-life of ractopamine in cattle. Further, one day of removal resulted in ractopamine residues being below the limit of detection in muscle and fat of pigs fed 18 mg of ractopamine/kg of feed for 28 d (Qiang et al., 2007). The duration of time needed for metabolic processes stimulated by ractopamine to return to baseline, and the means by which weight gain is reduced by cattle (or swine) after removing ractopamine relative to control cattle awaits discovery.

## CONCLUSION

Previous data from voluntary removal of ractopamine demonstrate that the added weight gain by pigs fed ractopamine is completely offset by 10 to 14 d of removal, and the findings of the current study indicate that the consequences of voluntary removal of ractopamine up to 8 d before slaughter were a linear decrease in live BW gain, poorer gain efficiency, and numerically lighter carcass weight. Known biology of ractopamine pharmacokinetics in cattle is supportive of the growth response observed by ractopamine removal in the present study. These data can be used by cattle feeders to generate economic estimates of the costs of removing ractopamine from finishing diets.

*Conflict of interest statement:* This study was conducted under contract with the principal investigator, Johnson Research, LLC. No Elanco employees were directly involved in the execution of the study activities.
